# Evidence of Heritability in Prebiotically Realistic Membrane-Bound Systems

**DOI:** 10.3390/life14030284

**Published:** 2024-02-20

**Authors:** Tymofii Sokolskyi, Pavani Ganju, Ronan Montgomery-Taylor, David A. Baum

**Affiliations:** 1 Wisconsin Institute for Discovery, University of Wisconsin-Madison, Madison, WI 53715, USA; sokolskyi@wisc.edu (T.S.);; 2 Department of Botany, University of Wisconsin-Madison, Madison, WI 53706, USA

**Keywords:** vesicles, origins of life, evolution, heritability, membranes

## Abstract

The vesicles of short chain amphiphiles have been demonstrated to grow and divide. Here, we explored whether vesicle populations show evidence of heritability. We prepared 1:1 decanoic acid:decylamine vesicles with or without a detergent and in either water or prebiotic soup, a mixture of compounds that might have been present on early Earth. The mixtures were subjected to transfer with dilution, where, after 24 h of incubation (one generation), we transferred 10% of the mix into a 90% volume of a fresh vesicle-containing solution. This was continued for 30 generations. Samples with a history of transfers were compared to no-transfer controls (NTCs), initiated each generation using the same solutions but without 10% of the prior generation. We compared the vesicle size distribution and chemical composition of the transfer samples and NTCs and compared their fluorescence signals in the presence of Nile Red dye. We observe changes in the vesicle size but did not detect differences in the chemical composition. In the samples with detergent and soup, we observed irregular changes in the Nile Red fluorescence, with a tendency for parent and offspring samples to have correlated values, suggestive of heritability. This last result, combined with evidence of temporal autocorrelation across generations, suggests the possibility that vesicles could respond to selection.

## 1. Introduction

Cellular encapsulation is the main mechanism by which extant life maintains concentration gradients and resists environmental stressors, making compartmentalization a key step in the origin of life [1,2]. The existence of compartments can also facilitate the emergence of adaptive dynamics by constraining parasitic chemical processes [3]. Some models imagine that the first evolvable life was already membrane-bound [2], whereas others imagine encapsulation arising from an earlier mode of spatial organization, for example, rock pores [4], mineral surfaces [5,6], aerosols [7], coacervate droplets [8,9] or micelles [10]. Whatever the primordial mode of compartmentalization, the cellular habit of all known life shows that vesicles with membranes composed of amphiphilic molecules must have originated very early. 

The prebiotic availability of amphiphiles has been questioned [11], but there is evidence to suggest that short- and medium-chain fatty acids were available on early Earth [12,13]. These would have formed during serpentinization [14] or have been delivered by extraterrestrial bodies such as meteorites [15,16] and comets [17]. 

Vesicles composed of fatty acids can grow by accreting amphiphile molecules and subsequently dividing [18]. This results in autocatalysis, which may help explain the discovery that simple vesicles can have a kind of collective heritability via the “matrix effect” [19]. The matrix effect arises when a small “seed” population of homogeneously sized vesicles is mixed with a much larger population of heterogeneously sized particles and causes the combined population to acquire a narrow size distribution, similar to the seed population. The underlying mechanism is thought to be a combination of template-directed nucleation and vesicle division dynamics [20,21]. In addition to the matrix effect, the replicating amphiphile particles, whether micelles or vesicles, can pass information to the following generations, encoded in the composition of their amphiphiles and/or associated compounds [10,22]. Such compositional heritability may allow for natural selection and has been used to suggest that vesicles are capable of adaptive evolution without the need for informational polymers [23,24]. 

Our research deploys a recursive seeding (RS) experimental paradigm [25], which resembles the experimental evolution protocols for microbial populations (e.g., [26]). In a microbial context, evolution is possible because alternative genotypes can self-replicate, allowing populations to transition to new state as a result of mutations, succeeded by drift or selection. We aimed to see whether analogous phenomena might be observed in vesicle populations by imposing RS on vesicles populations made from short chain amphiphiles, with or without a detergent or a mixture of organic compounds that might have been present on early Earth (“prebiotic soup”). The capacity for evolution would be supported by showing that there are vesicle traits that change systematically over the course of an RS experiment [27]. Additionally, the RS protocol allows us to look for evidence that the trait values in parent and offspring vials are correlated, which would imply some notion of heritability. As such, heritability can be quantified as the fraction of the variation in an offspring generation that can be predicted given the parents. 

To characterize populations of vesicles in the course of 30 generation of RS, we measured Nile Red fluorescence, which is known to be sensitive to multiple vesicle properties like size, shape, and lamellarity. We also estimated the particle size distributions using dynamic light scattering (DLS) and tracked the overall chemical composition using Liquid Chromatography–Mass Spectrometry (LC/MS). The chemical composition remained constant, but we observed temporal variation in the Nile Red fluorescence and vesicle size in the presence of a detergent. We also detected evidence of heritability, measured using linear regression of the parent and offspring trait values and temporal autocorrelation of the Nile Red fluorescence across generations, but only in the presence of detergent and prebiotic soup. Our findings demonstrate the value of the recursive seeding experimental paradigm and suggest the possibility that simple vesicles, interacting with prebiotic soup, have the potential to respond to selection.

## 2. Materials and Methods

### 2.1. Vesicle and Solution Preparation

The vesicles studied here were made of an equimolar mixture of decanoic acid:decylamine (DA:DN). These prebiotically plausible amphiphiles were chosen due to their ability to produce vesicles under high salt concentrations [28]. For our experiment, we incubated vesicles in two different solvents, water and “enriched prebiotic soup” (EPS). EPS is a complex solution consisting of amino acids, nucleobases, cofactors, carboxylic acids, and salts adjusted to a pH of 7 (Table S1), based largely on previous chemical ecosystem selection experiments [16,29].

To make the vesicles, decanoic acid (DA; A765305, VWR International, Hampton, VA, USA) and decylamine (DN; D2404, Sigma Aldrich, St. Louis, MO, USA) were dissolved in 1 mL of chloroform to a final concentration of 100 mM each. A thin film of amphiphiles was generated by evaporating the chloroform under a stream of nitrogen. Then, the films were hydrated with 10 mL of either Nanopure water (Thermo Fisher Scientific, Waltham, MA, USA) or the EPS stock solution to give a final concentration of 10 mM of each amphiphile. Subsequently, the solutions were sonicated for 10 min using an FS30 sonicator (Fisher Scientific, Hampton, VA, USA). A 10 min sonication was chosen based on preliminary data showing that the Nile Red fluorescence, which is sensitive to lamellarity, stabilized after 6 min of sonication (Figure S1A). After sonication, the samples contained vesicles primarily between 100 and 500 nm (Figure S1B). 

A subset of vesicle samples was incubated for 24 h with the detergent Triton X-100 (BP151500, Fisher Scientific, Hampton, VA, USA), which was added immediately prior to incubation to a final concentration of 0.05%. At higher Triton concentrations, the only particles detected in our analyses were ~10 nm Triton micelles (see Figure S6A). Despite being an unlikely prebiotic compound, Triton X-100 was included since it increases the vesicle size heterogeneity at low concentrations, is non-ionic, and can promote vesicle growth and division [30]. 

### 2.2. Recursive Seeding Design

**Figure 1:**
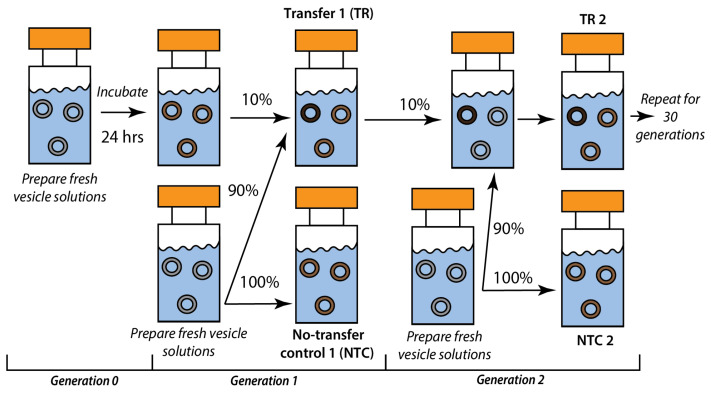
Scheme of the recursive seeding experimental design.

We used a recursive seeding (RS) protocol where 10% of the products of the first incubation were introduced as a “seed” into a fresh population of vesicles in the next generation (Figure 1). This procedure was repeated for 30 generations, where we compared the samples that experienced transfers in their history (TRs) to no-transfer controls (NTCs). NTCs control for differences in reagents and conditions across generations; the same vesicle solutions assembled in that generation were used, and they were incubated in identical conditions but did not receive a transfer from the previous generation. 

The RS experiment was implemented for four treatment categories: (1) EPS, DA:DN; (2) EPS, DA:DN, Triton; (3) water, DA:DN; (4) water, DA:DN, Triton. The experiment was conducted with 12 replicates per category per generation in 96-well plates (1185V32, VWR International, Hampton, VA, USA) sealed with plastic coverings (15036, VWR International, Hampton, VA, USA) and incubated at 25 °C in the dark in a 100 rpm shaking incubator. For the TRs, 30 μL of the respective TR from the previous generation was added to 270 μL of the fresh vesicle solution. For the NTC solutions, to control for pipetting, 30 μL of the fresh solution was added to a separate batch of 270 μL of the same fresh solution. At the end of the incubation for each generation, the plates were stored in a −80 °C freezer and only thawed immediately prior to conducting the analyses. All samples subject to the same kind of analysis were thawed an equal number of times: one thaw for DLS and two for Nile Red fluorescence.

### 2.3. Nile Red Fluorescence Measurements

Nile Red (NR) is a dye that fluoresces in non-polar environments with a fluorescence intensity that is sensitive to a variety of factors [31]. To measure the NR fluorescence, we transferred the samples into black 96-well plates (37000-550, Thermo Fisher Scientific, Waltham, MA, USA) and added NR (ENZ-52551, Fisher Scientific, Hampton, VA, USA) to each well from a 1 mM stock solution in ethanol to a final concentration of 0.01 mM. The plates were then placed in the dark at 25 °C in a 100 rpm shaking incubator for 1 h. Subsequently, the fluorescence emissions at 610, 640, and 660 nm were quantified using a BioTek Synergy HT4 microplate spectrophotometer (Agilent Technologies, Santa Clara, CA, USA) with excitation at 480 nm. The fluorescence intensity was compared between the TRs and NTCs using a two-tailed heteroscedastic *t*-test. 

To look for evidence of heritability, we conducted linear regression for each TR sample in one generation versus its descendant sample in the next generation. To assess whether the correlation coefficients were more likely to be positive than negative for a given treatment, we used the non-parametric Wilcoxon signed-rank test. To assess the longer-term patterns, temporal autocorrelation was assessed using the statsmodels module in Python [32]. This method determines whether, looking across all generations, the mean value of a given generation is correlated with that found in the preceding generation (lag = 1), the one before that (lag = 2), the one before that (lag = 3), etc. We only considered lags 1–5 since longer lags are difficult to interpret. 

To help with the interpretation of our results, we also determined the effect of 24 h of incubation on the NR fluorescence of the Triton-containing samples. We set up 32 replicates and then measured half of the samples at the start and half at the end of the 24 h incubation. 

### 2.4. Dynamic Light Scattering

The particle size distributions were inferred using dynamic light scattering (DLS) and a Zetasizer Nano (Malvern Pananalytical, Malvern, UK) with 175° backscattering at 25 °C with 3 replicate measurements per sample. The time-resolved correlation function was converted into a hydrodynamic radius versus intensity distribution using the Malvern General Purpose (GP) non-negative least squares distribution fit algorithm with a 0.01 regularizer and 70 size classes [33,34]. As this analysis takes about 4 h per treatment category in a cuvette reader for a single generation, we analyzed different generations on separate days but always analyzed the TRs and their corresponding NTCs at the same time. To reduce the possibility that the time of analysis could cause artifactual differences between the TR and NTC samples, paired samples were analyzed in an alternate manner (e.g., TR11, NTC11, TR12, NTC12, etc.). Each sample was diluted tenfold in a cuvette with Nanopure water immediately prior to measurement to increase the DLS resolution, with the exception of the EPS samples without Triton, where the DA:DN samples were diluted 100-fold to minimize the interference from a strong peak at around 1 nm, likely representing some kind of precipitate. 

### 2.5. LC/MS Analyses

For the samples not containing Triton (which is incompatible with LC/MS), we analyzed the bulk chemical composition using an untargeted metabolomics approach with reverse-phase Ultra-Performance Liquid Chromatography coupled with tandem mass spectrometry (UPLC–MS/MS). The vesicle samples were thawed and immediately diluted 1:10 in LC-grade methanol (A4564, Fisher Scientific, Hampton, VA, USA) and incubated at 25 °C with 100 rpm shaking for 1 h prior to loading onto the LC/MS instrument. We used a Thermo Vanquish UHPLC (Thermo Fisher Scientific, Waltham, MA, USA) with a C18 column (Agilent, Santa Clara, CA, USA), with 5 μL of the samples injected and then eluted in a linear gradient mixture from 0.1% v/v formic acid in water (47146-M6, Thermo Fisher Scientific, Waltham, MA, USA) to 0.1 % v/v formic acid in acetonitrile (47138-K7, Thermo Fisher Scientific, Waltham, MA, USA) over 14 min. To minimize order effects, the samples were run with the first replicate of all treatments preceding the second replicate of all treatments, and so on. 

The UPLC was coupled to a Thermo Q-Exactive Plus Orbitrap MS (Thermo Fisher Scientific, Waltham, MA, USA). The full MS-SIM spectra for each of the replicates were collected for 10 minutes in positive mode over a scan range of 50–750 m/z, with the resolution set to 70,000. Fragmentation data were collected only for pooled samples for each treatment category, with full MS followed by data-driven MS2 analysis (dynamic exclusion of 4 s; intensity threshold of 10^5^; resolution 17,500; isolation window 1 m/z; stepped collision energies of 20, 50, and 100 eV). The data files were processed in Compound Discoverer^TM^ version 3.3 (Thermo Fisher Scientific, Waltham, MA, USA) using the default untargeted metabolomics workflow with 16 added databases (Acros Organics, Alfa Aesar, Alfa Chemistry, BioCyc, the Cambridge Structural Database, CAS Common Chemistry, ChemBank, DrugBank, FDA, the Human Metabolome Database, KEGG, MassBank, Merck Millipore, MeSH, NIST, the NP Atlas) and the pooled samples set to “identification only”. Lists of the identified compounds and their relative abundances were extracted, and the differences between the TRs and NTCs were evaluated using heteroscedastic *t*-tests with Bonferroni alpha correction, while principal component analysis plots were produced using Compound Discoverer^TM^.

## 3. Results

### 3.1. Nile Red Fluorescence Measurements

In the samples without Triton, the NR fluorescence at the maximum emission wavelength of 640 nm (I_640_) was minimal, and we observed a lack of differences between the TRs and NTCs (Figure S2). The I_640_ values for the NTC Triton samples were much higher (Figure S3), having increased significantly over the 24 h of incubation (Figure S4). There was a high level of variability over generations, suggesting some unidentified source of variation in the vesicle preparation. However, since the same batch of vesicles was used for the TR and NTC samples, this variation should not invalidate TR:NTC comparisons within generations. 

**Figure 2:**
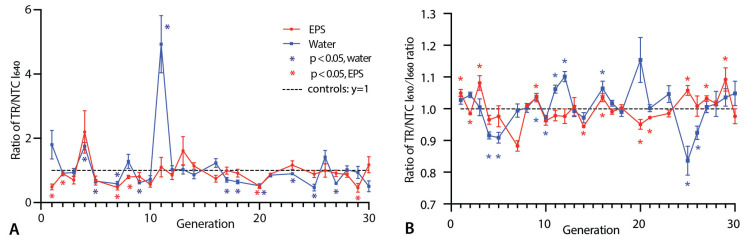
Change in NR fluorescence over generations for samples containing Triton: (**A**) fluorescence intensity with 640 nm emission (I_640_); (**B**) ratio of intensity at 610 nm to 660 nm (I_610_/I_660_). The TR/NTC ratio for each measured generation is shown. Stars mark significant (*p* < 0.05) differences between TRs and NTCs as determined using heteroscedastic *t*-tests on raw TR and NTC values. Error bars are standard errors (12 replicates per measurement).

The samples containing Triton demonstrated a significant difference (*p* < 0.05) in the NR fluorescence for the TRs relative to the NTCs for 11 out of 25 measured generations in water and 6 in the EPS (Figure 2A; Table S2). Significant differences were observed as early as generation 1 in the EPS samples. Interestingly, the TRs had a significantly lower NR fluorescence in generation 1 despite being seeded with a sample (from generation 0) that should, based on the 24 h incubation data, have started with a higher fluorescence (Figure S4). Of the 17 generations with significant differences in either condition, 15 had lower TR than NTC fluorescence. For reasons that are unclear, generation 11 in water was an outlier both in terms of the magnitude and direction of the TR/NTC ratio.

In addition to I_640_, we calculated the ratio of intensity at 610 nm to 660 nm (I_610_/I_660_), which has proven to provide useful information on the polarity of the chemical environment of NR [35,36]. In both the EPS and water samples, the I_610_/I_660_ values varied greatly between the TR and NTC samples, being significantly (*p* < 0.05) different in 11 generations of the EPS lineage and 10 generations in water (Figure 2B). In 7 of the 11 EPS generations, the I_610_/I_660_ value is higher in the TRs, contrasting with 4 out of 10 generations in the water samples. 

To determine whether our samples exhibited heritability, we calculated Pearson’s correlation coefficients for the I_640_ values between the parent and offspring samples within the TR lineages. The results of this test are summarized in Table 1. For the EPS samples, we find significant (*p* < 0.05) positive correlation values for four generations in the TRs and one generation in the NTCs. For the water samples, the correlation between adjacent generations is mostly negative, with two generations with significant correlation in the NTCs and three generations in the TRs. Using the non-parametric Wilcoxon’s signed-rank test to evaluate the null hypothesis that r-values are as likely to be positive as negative, returns *p* = 0.056 for all the EPS TR pairs or *p* = 0.040 when samples more than one generation apart (i.e., red values in Table 1) are dropped. While not corrected for multiple tests, this result provides preliminary support for there being some degree of heritability of NR fluorescence (I_640_) in the presence of EPS. No evidence of heritability was seen for I_610_/I_660_. 

Given the evidence of heritability of I_640_, we also explored the longer-term patterns in the TR:NTC ratios (Figure 2A) over generations using autocorrelation analysis. We find a significant positive autocorrelation for lags 1 and 2 for the EPS samples (Figure S5A,B). This means that the mean value from a given generation is more similar to the prior two generations than expected according to chance. Lags 3 and 4 also showed positive autocorrelation, but this was not significant at lag 3. There was no such pattern of positive autocorrelation for the water samples; indeed, there was a significant negative autocorrelation for lag 2 (Figure S5A,B). Analyses based on the raw TR values found significant positive autocorrelation at lag 2 in the EPS (Figure S5E) but the NTCs, which have no transfer history, also showed a significantly positive autocorrelation at lag 3 (Figure S5C).

### 3.2. Vesicle Size Measurements

We monitored the vesicle radius change over the course of the experiment using DLS. The freshly made DA:DN vesicles show a single peak between 100 and 200 nm that is robust to pH (Figure S6A). Similarly, the pure Triton solutions showed a single peak at 10 nm, presumably representing micelles (Figure S6B). 

**Figure 3:**
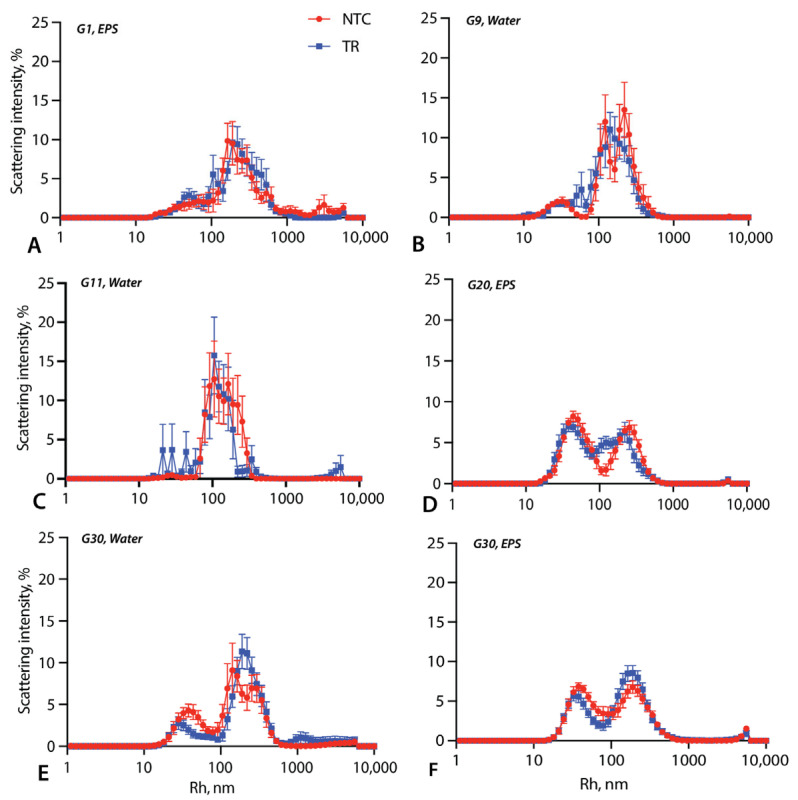
Particle size distributions for samples containing Triton. DLS intensity/hydrodynamic radius distributions are shown for selected generations of EPS ((**A**) generation 1, (**D**) generation 20, (**F**) generation 30) and water ((**B**) generation 9, (**C**) generation 11, (**E**) generation 30). Error bars are standard errors across the 10 replicate samples. Data for other generations are shown in Figure S7.

Although the size distributions varied considerably, the Triton-containing samples often contain particles of two size categories, with modes around 30 nm and 200 nm (Figure 3 and Figure S7). In some generations, for example, generations 1 and 30 in the EPS and generation 9 in water, the TRs and NTCs had almost identical size distributions (Figure 3A,B,E). In other generations, the TRs and NTCs differed. For example, in generation 11 in water, the TR samples have an additional peak between 10 and 50 nm that is not seen in the NTCs (Figure 3C). This may partially explain why this generation was an outlier for I_640_ (Figure 2A). In generation 20 in the EPS, the TRs contain more vesicles in the 90–150 nm size range than the NTCs (Figure 3D). The NTCs in generation 30 in water contained more particles <100 nm (Figure 3E).

**Figure 4:**
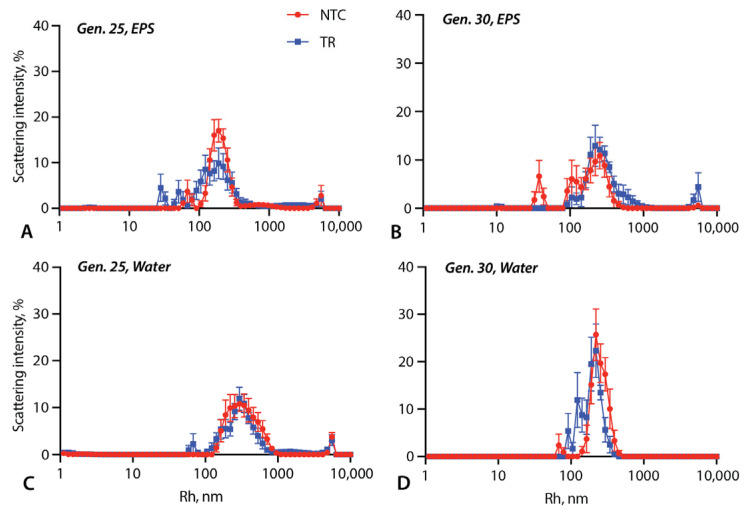
DLS results for samples without Triton. DLS intensity/hydrodynamic radius distributions are shown for selected generations: generation 25, EPS (**A**) and water (**C**) and generation 30, EPS (**B**) and water (**D**). Error bars are standard errors across 10 replicate samples.

The samples without Triton showed similarly diverse size distributions (Figure 4) and, again, differences between the TRs and NTCs were observed in some generations. In generation 25 in the EPS, the TR samples included more particles <100 nm (Figure 4A), whereas there was an excess of particles >1000 nm in the generation 30 TRs (Figure 4B). In water, in both generations 25 and 30, the TR size distributions included small peaks below 100 nm that were absent in the NTCs (Figure 4C,D).

### 3.3. Chemical Composition of the EPS Samples without Triton

**Figure 5:**
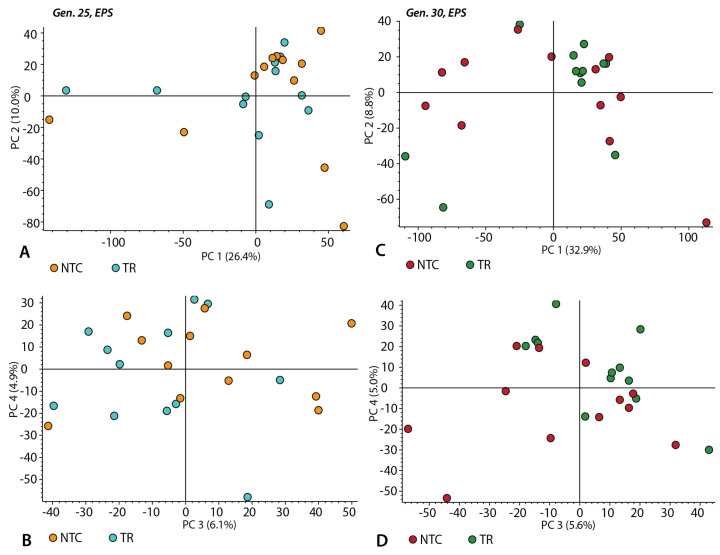
Principal component analysis of the relative abundance of spectral features identified using Compound Discoverer for generation 25 EPS (**A**,**B**) and generation 30 EPS (**C**,**D**). (**A**,**C**) are plots of principal component (PC) 1 vs. PC2; (**B**,**D**) are PC3 vs. PC4 (12 replicates per category).

To understand whether the chemical composition of the EPS is affected by the transfer history, we analyzed the TR and NTC samples using LC/MS in generations 25 and 30. Since Triton is incompatible with our LC/MS protocol, only samples without detergent were analyzed. The samples contained several thousands of spectral features identified using Compound Discoverer, with less than 5% being significantly different at *p* < 0.05. This result is consistent with the TR and NTC samples being compositionally identical. Consistent with this, principal component analysis of the total chemical composition of the generation 25 (Figure 5A,B) and 30 (Figure 5C,D) samples shows a complete overlap between the NTCs and TRs. 

Since both DA and DN could be detected using LC/MS, we assessed whether these differed measurably between the TR and NTC samples. No significant differences were detected (Figure S8).

## 4. Discussion

### 4.1. Limitations of the Study

Although we attempted to standardize the setup for each generation as much as possible, we still observed marked variation between the NTCs from different generations, especially for NR I_640_ (Figure S3). It is unclear whether the intergenerational variation is a result of minor differences in the solution preparation or subtle changes in temperature or other environmental variables. One potential source of noise is variation in the duration of freezing. However, although the time that the samples spent in a frozen state varied among generations, all samples that were compared to each other (e.g., EPS samples from two different generations) were thawed an equal number of times prior to analysis, which is important because freezing and thawing change the membrane dynamics [37]. Moreover, the TR and NTC samples from a given generation were always frozen and thawed at the same time.

### 4.2. Explanations of the Observed Patterns

In this study, we measured the vesicle size, chemical composition, and Nile Red fluorescence over the course of multiple generations of RS. Except for the chemical composition, we observed multiple cases of significant differences between the TR and NTC samples. However, no feature consistently increased or decreased from generation 1 to 30. For example, although I_640_, which was measured in 25 out of 30 generations, showed a tendency to have lower values for the Triton TRs than their corresponding NTCs, this ratio (and the underlying raw values) fluctuated rather than steadily increased or decreased. 

The observation of a large number of generations in which I_640_ for the Triton samples is significantly lower in the TRs than in the NTCs cannot be explained by the transfer protocol itself. On the contrary, since I_640_ is higher after the vesicles have been incubated for 24 h (Figure S4), we might have expected the TR samples, which receive 90% fresh reagent and 10% previously incubated sample, to have a higher I_640_ than the NTC samples, which received only fresh reagents. The fact that we saw the opposite pattern suggests that some kind of non-linear vesicle dynamics are triggered by seeding. 

NR is a dye that fluoresces in non-polar environments due to the twisted intramolecular charge transfer (TICT) between the quinone and diethylamino groups [38] and exhibits different emission peaks based on the solvent [39]. Since the NR fluorescence intensity is proportional to the number of NR molecules undergoing TICT, it provides a measure of the abundance of non-polar regions containing NR, which can be affected by many factors, including the particle size, lamellarity, area/volume ratio, and particle shape [31,40]. Such complex photophysics is beneficial to our study, as it allows us to track multiple vesicle traits at once without focusing on one specific property. 

Although it is possible for changes in I_640_ to result from changes in the membrane composition, this is probably not relevant here since we failed to detect chemical composition differences between samples. Likewise, because we sonicated the samples for 10 min, whereas just 6 min of sonication is sufficient to stabilize I_640_ (Figure S1), changes in lamellarity cannot easily explain the TR/NTC differences. Instead, the differences in I_640_ are probably due to changes in the particle size distributions, including the abundance of micelles. 

Pure Triton produces 10 nm micelles (Figure S6B). However, in the presence of DA:DN lipids, peaks of this size category are absent (Figure 3). This indicates that while micelles may be present, they must be larger (>30 nm) and include DA and/or DN in addition to Triton. The samples with the DLS peaks of the smallest size in the TRs are from generation 11 in water (Figure 3C). It may therefore not be a coincidence that this generation is an outlier in having a significantly greater I_640_ value in the TR than NTC (Figure 2A,B). However, there is no consistent correlation between the micelle abundance and reduced fluorescence measurements in the TRs. 

Interestingly, in addition to I_640_, the I_610_/I_660_ ratio is also significantly different between the TRs and NTCs in multiple generations. I_610_/I_660_ can be used to infer the micropolarity of a system, with greater values (a stronger blue shift in emissions) corresponding to greater burial of the NR molecules into hydrophobic structures, such as droplets or bilayers [35,36]. The differences in the micropolarity between the TRs and NTCs suggest changes in the abundance of different particle types, only some of which were detectable using the DLS analyses. It is difficult to determine which specific types of particles (vesicles, micelles, droplets, etc.) contribute to this dynamic. Nonetheless, we found that micropolarity changes significantly over the course of the experiment and is sensitive to a transfer history.

In addition to changes in the relative frequency of different aggregates, changes in vesicle size could explain the lower I_640_ values in the TRs than in the NTCs. As shown in Figure 3, the TRs and NTCs differed in their particle size distributions in several generations. For example, in generation 20 in the EPS, there were more larger vesicles in the TRs (Figure 3D), which is consistent with the significantly lower I_640_ value in that generation (Figure 2A). However, the size distributions of generation 30 in water showed a similar pattern without a significant difference in I_640_ (Figure 2A and Figure 3E). Likewise, generations 1 in the EPS and 9 in water display a significantly lower I_640_ fluorescence in the TRs but no major difference in the DLS results (Figure 2A and Figure 3A,B). Thus, while the vesicle size distributions and the proportion of micelles likely contribute to different I_640_ levels, it is likely that multiple factors were responsible for the TR–NTC differences, of which changes in shape are worth exploring further in the future. Overall, therefore, it is difficult to assign the observed I_640_ dynamics to changes in one specific vesicle property. 

### 4.3. Heritability in Vesicle Populations

Vesicles of short chain amphiphiles are known to replicate [18], but it remains uncertain whether this replication process supports the generation of heritable variation that selection could act upon. Here, we established RS lineages and looked to see whether there was a tendency for samples to inherit characteristics from their parents in the preceding generation. This would be indicated by positive parent–offspring correlations. Additionally, if mean trait values change over time, such heritability would predict a positive autocorrelation in the average trait values over generations. 

For the TRs in water, there is no consistent parent–offspring regression for I_640_, similar to the pattern seen for the NTCs: there are only 2–3 significant correlation coefficients, and these are positive as often as negative, with an average correlation coefficient close to zero. For the TRs in the EPS, however, there are four significant correlation coefficients, and all are positive. The average correlation over all generations in EPS was +0.15 (Table 1), which is significantly (or close to significantly) greater than zero depending on whether one includes comparisons that are two generations apart (due to missing samples). Although replication with larger sample sizes is called for, these results suggest the possibility that populations of prebiotically plausible vesicles interacting with EPS are able to express heritable variation upon which selection might be able to act. 

This analysis of heritability suggests the possibility of non-genetic inheritance in the EPS–Triton samples but not in the other treatments. If so, and there was also a non-genetic analog of mutation, selection (or mutation bias) could drive directional changes in vesicle populations over generations. Alternatively, we might expect traits to drift over generations by chance. In either case, we there should be temporal autocorrelation, where the mean value of a given generation is more similar to its preceding and following generations than to random generations in the RS sequence. 

Given that raw I_640_ is sensitive to the mix of vesicles made in each generation, which varied greatly (as seen in the large fluctuations even in the NTCs), autocorrelation analysis is best conducted on the TR/NTC ratio. At lag 1, we found a significantly positive autocorrelation of ~0.12 in the EPS–Triton samples but a score of ~0 for the water samples. The positive temporal autocorrelation in EPS–Triton samples was also seen at lags two, three, and four (0.13, 0.09, and 0.30, respectively). These results warrant replication, but taken together, imply the possibility that the EPS–Triton samples may have the capacity for non-genetic evolution. 

The matrix effect offers a potential mechanism of heritability that does not require template-based polymerization. However, more experiments and theoretical models would be needed to conclude that this phenomenon plays a role in vesicle heritability. Given that the effect is only seen in the presence of EPS, compositional inheritance [23,24] would to be a more plausible explanation for heritability in this case. 

Interactions of amphiphile assemblies with their chemical environments were emphasized by the Lipid World hypothesis [22]. Models showed that micelles composed of diverse amphiphiles, some of which have catalytic activities, can demonstrate heritability and a response to selection [10,24]. Similar phenomena could readily occur in vesicle populations due to interactions among amphiphiles [13,28] and/or amphiphile-induced catalysis of other reactions [41,42,43]. Additionally, stochastic encapsulation of solutes, such as the “all-or-nothing” encapsulation of proteins [44], could contribute to the heterogeneity of populations and could result in heritability provided that the chemicals trapped in the vesicles show autocatalysis. The models of evolution for systems with compositional inheritance have drawn criticism due to their low replication accuracy [24]. However, in complex systems with multiple distinct components of the membrane and the vesicle lumen, there may be interactions that constrain variation. It is possible, for example, that interactions between the compounds in the vesicle interior or exterior could stabilize vesicle states in a composition-specific manner [45,46]. 

RS experiments are structured in such a way that, if a certain type of vesicle arises that could propagate its characteristics through serial transfer more effectively than other vesicle types, that type would tend to increase in abundance. Thus, even without imposing conscious selection, the experimental paradigm is capable of inducing adaptive evolution. This may be an explanation for the lack of significant heritability of I_640_ in every generation since a response to selection often reduces heritability. 

Given the evidence of heritability of I_640_ even with a 10% transfer fraction, it would be exciting to conduct RS experiments while imposing artificial selection. For example, if there were a way to only seed each generation with those vesicles from the prior generation that resisted a certain environmental challenge, we could then see whether resistance to that challenge increased over generations. Adding such direct selection would have the benefit of predicting the direction of the selective response, which should allow for clearer and more definitive statistical tests. The work conducted here provides a valuable baseline for future research by defining the range of behavior that can be expected even in the absence of intentional selection. 

Despite its limitations, this study represents the first attempt to experimentally explore evolution-like dynamics in amphiphilic vesicles. We obtained tentative evidence of heritability and history dependence via temporal autocorrelation, which implies that short chain amphiphile vesicles interacting with complex organic mixtures may show heritability and be capable of adaptive evolution. Although this result warrants replication, ideally utilizing automation to increase the sample size and improve the replicability, our study illustrates the potential value of RS studies with different amphiphiles and prebiotic soup to shed light on prebiotic evolutionary mechanisms. 

## Figures and Tables

**Table 1:** Pearson’s correlation coefficients (r-values) for I_640_ between parent and offspring generations in the four treatments. Pairs of generations that are more than one generation apart, because the generation in between was not measured, are underlined. Correlation coefficients with an absolute value greater than 0.3 are in bold. Asterisks (*) mark *p* < 0.05. For EPS TR samples, nine of the ten coefficients over 0.3 are positive, including all four significant coefficients.

Generations	Water [NTC]	Water [TR]	EPS [NTC]	EPS [TR]
1–2	−0.18	−0.62 *	**0.43**	−0.09
2–3	0.12	0.01	0.24	0.65 *
3–4	0.00	−0.08	**−0.38**	−0.05
4–5	−0.25	**−0.41**	0.71 *	0.03
5–7	−0.11	0.02	**−0.51**	-0.29
7–8	**0.50**	−0.20	0.04	0.68 *
8–9	**−0.55**	−0.77 *	**0.36**	−0.02
9–10	**−0.50**	**−0.52**	0.17	−0.19
10–11	0.19	**0.42**	**0.30**	0.58 *
11–12	0.04	0.18	−0.01	**0.34**
12–13	−0.14	0.27	0.16	−0.07
13–14	0.01	0.22	0.18	0.17
14–16	**0.39**	−0.06	0.28	**0.47**
16–17	0.29	−0.18	0.06	0.28
17–18	**0.43**	**−0.43**	**0.31**	**0.37**
18–20	0.13	0.11	−0.19	−0.09
20–21	0.04	**−0.57**	−0.27	0.12
21–23	0.59 *	0.13	0.09	**0.31**
23–25	0.43	0.21	−0.22	−0.23
25–26	**−** 0.58 *	−0.04	**−0.52**	**0.32**
26–27	0.14	−0.23	**0.50**	0.64 *
27–28	−0.15	−0.23	−0.15	**−0.35**
28–29	0.24	−0.25	−0.09	0.07
29–30	−0.21	0.76 *	0.09	−0.04
Mean r	0.04	−0.09	0.07	0.15

## Data Availability

Publicly available datasets were analyzed in this study. This data can be found here: https://drive.google.com/file/d/152OdaDBc_2wNW9KTWdivjVoVksFu1tip/view?usp=sharing, accessed on 19 February 2024.

## Supplementary Materials

The following supporting information can be downloaded at https://www.mdpi.com/article/10.3390/life14030284/s1. Table S1: EPS composition; Table S2: NR fluorescence *p*-values comparing TRs and NTCs; Figure S1: Effect of sonication on vesicles; Figure S2: NR fluorescence of generation 25 samples with and without Triton; Figure S3: Raw NR fluorescence of samples with Triton; Figure S4: Impact of incubation on NR fluorescence; Figure S5: Autocorrelation of NR fluorescence; Figure S6: Size of vesicles and micelles; Figure S7: Additional size distributions of samples with Triton; Figure S8: Relative abundance of amphiphiles based on LC-MS.
