# Supplementary material for: Evidence of Heritability in Prebiotically Realistic Membrane-Bound Systems
Source: Life (Basel). 2024 Feb 20;14(3):284. doi: 10.3390/life14030284 (PMC10971454; doi:10.3390/life14030284)
Supplement: Supplementary file 1 [file life-14-00284-s001.zip › life-2816702-supplementary.pdf]

Supplementary Materials

Evidence of heritability in prebiotically realistic membrane-bound systems

Tymofii Sokolskyi[1,2], Pavani Ganju[1,2], Ronan Montgomery-Taylor[1,2], David Baum[1,2]

1 – Wisconsin Institute for Discovery, University of Wisconsin-Madison, Madison, WI 53715, USA

2 – Department of Botany, University of Wisconsin-Madison, Madison, WI 53706, USA

Enriched Prebiotic Soup (EPS) preparation

Compounds in concentrations listed in Table S1, with the exceptions of ammonium persulfate (APS) and sodium trimetaphosphate (TMP), were mixed in a glass beaker and then dissolved in 1L of water with stirring. Resulting solution was dispensed into 50 mL plastic tubes and stored at -20°C until use. Immediately prior to experimental use, EPS stocks were thawed, filtered through a 200 nm syringe filter (431215, DOT Scientific Inc., Burton, USA) and TMP and APS were added to appropriate final concentrations.

**Compound**	**Class**	**Vendor**	**CAS #**	**Final Concentration (mM)**
1,3-dihydroxyacetone	Triose	Fisher Scientific	96-26-4	0.32
2-aminobutyric acid	Amino Acid	Acros Organics	2835-81-6	0.08
acetic acid	Organic Acid	Sigma-Aldrich	?	0.32
acetoguanamine	Triazine	VWR	541-02-9	0.16
adenine	Nucleobase	Fisher Scientific	73-24-5	0.32
ammonium chloride	Salt/Redox Energy	Sigma-Aldrich	12125-02-9	20
ammonium persulfate**	Salt/Oxidant	IBI Scientific	7727-54-0	0.04
butyric acid (sodium salt)	Organic Acid	Fisher Scientific	156-54-7	0.08
cobalt(II) chloride anhydrous	Transition Metal Salt	BTC Chemicals	7646-79-9	0.001
copper(II) chloride dihydrate	Transition Metal Salt	Alfa Aesar	10125-13-0	0.001
cytosine	Nucleobase	Fisher Scientific	71-30-7	0.32
D-(-)-ribose	Monosaccharide	Fisher Scientific	50-69-1	0.16
D-(+)-xylose	Monosaccharide	Fisher Scientific	58-86-6	0.16

D-glucose	Monosaccharide	DOT Scientific Inc.	54-99-7	0.16
DL-arabinose	Monosaccharide	VWR	147-81-9	0.16
formic acid 90%	Organic Acid	Aqua Solutions	64-18-6	0.64
glycerol	Trihydric Alcohol	DOT Scientific Inc.	56-81-5	0.32
glycolic acid	α-Hydroxy Acid	Acros Organics	79-14-1	0.32
hydroxybutyric acid	β-Hydroxy Acid	Sigma-Aldrich	150-83-4	0.08
iminodiacetic acid	Dicarboxylic Acid Amine	VWR	142-73-4	0.16
L-alanine	Amino Acid	VWR	56-41-7	0.32
L-arginine	Amino Acid	Sigma-Aldrich	74-79-3	0.16
L-ascorbic acid	Reducing Agent/Cofactor	DOT Scientific Inc.	50-81-7	0.04
L-asparagine	Amino Acid	Acros Organics	5794-13-8	0.16
L-aspartic acid	Amino Acid	Alfa Aesar	56-84-8	0.32
L-cysteine	Amino Acid	DOT Scientific Inc.	52-90-4	0.16
L-glutamic Acid	Amino Acid	VWR	56-86-0	0.32
L-glutamine	Amino Acid	Sigma-Aldrich	56-85-9	0.16
L-glycine	Amino Acid	Sigma-Aldrich	54-40-6	0.32
L-histidine	Amino Acid	DOT Scientific Inc.	71-00-1	0.08
L-isoleucine	Amino Acid	DOT Scientific Inc.	73-32-5	0.16
L-leucine	Amino Acid	DOT Scientific Inc.	61-90-5	0.32
L-lysine	Amino Acid	DOT Scientific Inc.	657-27-2	0.32
L-methionine	Amino Acid	DOT Scientific Inc.	63-68-3	0.08
L-phenylalanine	Amino Acid	DOT Scientific Inc.	63-91-2	0.16
L-proline	Amino Acid	Alfa Aesar	147-85-3	0.16
L-serine	Amino Acid	DOT Scientific Inc.	56-45-1	0.32
L-threonine	Amino Acid	DOT Scientific Inc.	72-19-5	0.32
L-tryptophan	Amino Acid	DOT Scientific Inc.	73-22-3	0.08

L-tyrosine	Amino Acid	Amresco	60-18-4	0.16
L-valine	Amino Acid	DOT Scientific Inc.	72-18-4	0.32
lactic acid 88% solution	α-Hydroxy Acid	Fisher Scientific	50-21-5	0.16
magnesium chloride	Sea Salt	DOT Scientific In.	7791-18-6	50
N-ethanolamine	Amino Alcohol	Sigma-Aldrich	141-43-5	0.08
N-methylalanine	Amino Acid	Sigma-Aldrich	3913-67-5	0.08
N-methylglycine	Amino Acid Derivative	Acros Organics	107-97-1	0.16
N-methylurea	Amide	Fisher Scientific	759-73-9	0.08
nicotinamide	Cofactor	DOT Scientific Inc.	98-92-0	0.08
potassium chloride	Sea Salt	VWR	7447-40-7	10
propionic acid	Organic Acid	Fisher Scientific	79-09-4	0.16
pyruvic acid	α-Keto Acids	Fisher Scientific	127-17-3	0.08
R-pantetheine	Cofactor	Sigma-Aldrich	496-65-1	0.04
sodium bisulfite	Reducing agent	Ward's	7631-90-5	0.08
sodium chloride	Sea Salt	Fisher Scientific	7647-14-5	500
sodium molybdate	Transition Metal Salt	Strem Chemicals	10102-40-6	0.001
sodium nitrate	Salt	Sigma-Aldrich	7631-99-4	20
succinic acid	Dicarboxylic Acid	Sigma-Aldrich	110-15-6	0.08
succinonitrile	Nitrile	VWR	110-61-2	0.16
thymine	Nucleobase	Fisher Scientific	65-71-4	0.32
trisodium trimetaphosphate**	Phosphate Source	Sigma-Aldrich	7785-84-4	0.1
uracil	Nucleobase	VWR	66-22-8	0.32
urea	Amide	IBI Scientific	57-13-6	0.16
zinc(II) chloride	Transition Metal Salt	Sigma-Aldrich	7646-85-7	0.001

β-alanine	Amino Acid	Tokyo Chemical	107-95-9	0.32
iron (II) chloride	Transition Metal Salt	Alfa Aesar	7758-94-3	0.001
manganese (II) sulfate monohydrate	Transition Metal Salt	Sigma-Aldrich	7487-88-9	0.001

Table S1. EPS composition. ** indicates compounds that were added immediately prior to use (TMP and APS).

Effect of sonication time on vesicle size and NR fluorescence. To test whether vesicle lamellarity is a contributing factor to changes in NR fluorescence and size, we measured vesicle solutions in water without Triton, sonicated for different times. Since sonication time is expected to be inversely proportional to lamellarity (Maurer et al., 2018), we predicted a decline in NR fluorescence with sonication. As predicted, NR fluorescence drops after ~4 minutes of sonication and remains stable for the duration of the time-course (**Fig. S1, A**). Similarly, vesicle size distribution of unsonicated vesicles suggests a larger size than vesicles sonicated for 10 minutes (**Fig. S1, B**).

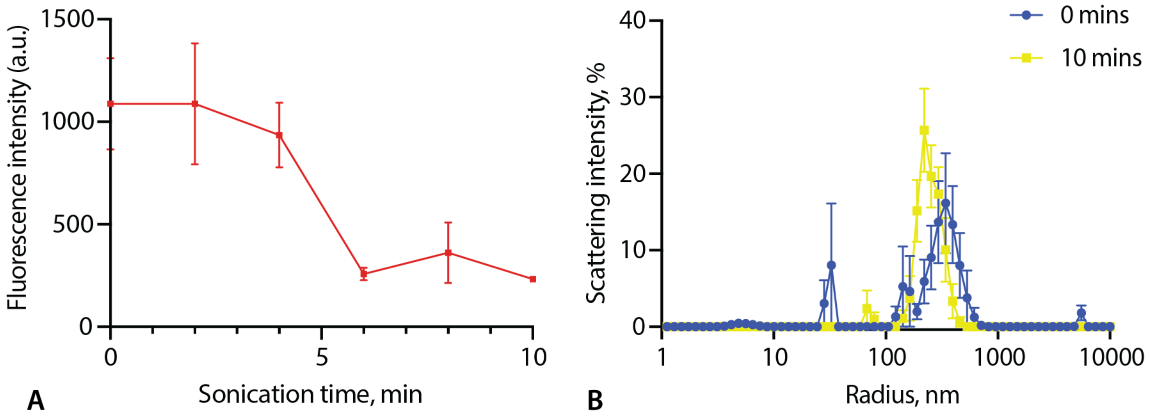

Figure S1. Results of the 10 minute sonication test on vesicles composed of 20 mM 1:1 DA:DN in water. **A** – Nile Red fluorescence intensity over sonication time; **B** – size distributions of unsonicated and sonicated vesicles. Error bars are standard error (12 replicates for **A**, 3 replicates for **B**).

Table S2. p-values derived from a two-tailed heteroscedastic t-test comparing raw NTC and TR NR fluorescence intensity values of samples containing Triton X-100 in either EPS or water.

Generation	EPS	Water
1	0.00055171	0.153476769
2	0.00491489	0.579675521
3	0.16115933	0.6836678
4	0.11314095	0.002685796
5	0.21314719	0.000592203
7	0.00855078	0.007956636
8	0.009686	0.381839385
9	0.17410591	2.91172E-07
10	0.17949402	0.095197113
11	0.76135622	0.001002305
12	0.41611784	0.866752562
13	0.21922503	0.886798447
14	0.31832474	0.379641724
16	0.09234096	0.286683154
17	0.9716847	0.020788254
18	0.55434033	0.001030556
20	0.00183984	0.044239599
21	0.07552436	0.099381047
23	0.27278224	0.049111401
25	0.39111247	0.007031338
26	0.99117549	0.104085514
27	0.59941244	0.000388879
28	0.41574193	0.903164937
29	0.00295851	0.793949012
30	0.59977185	0.115396303

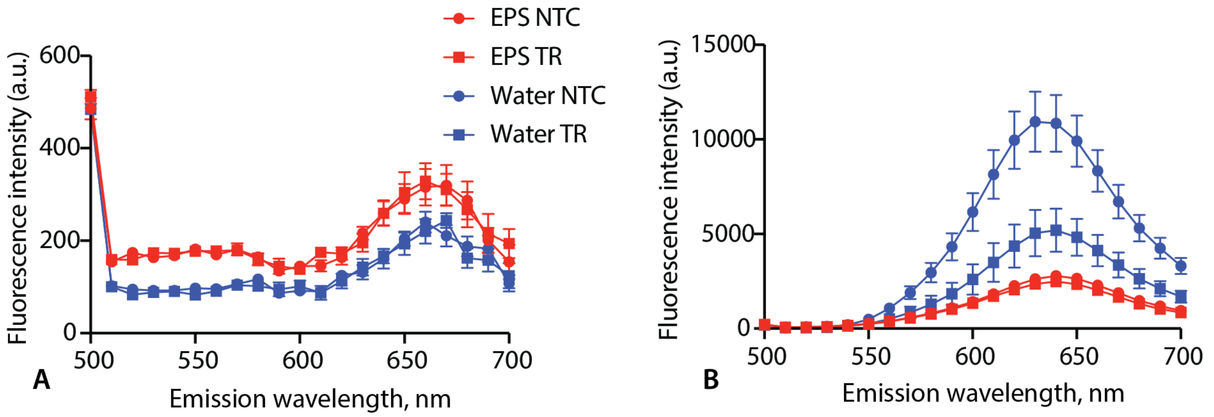

Figure S2. Comparison between Nile Red fluorescence emission spectra with 480 nm excitation wavelength for samples without (**A**) and with (**B**) Triton X-100 at generation 25. Note extremely low fluorescence of samples without the detergent. Error bars are standard error (12 replicates).

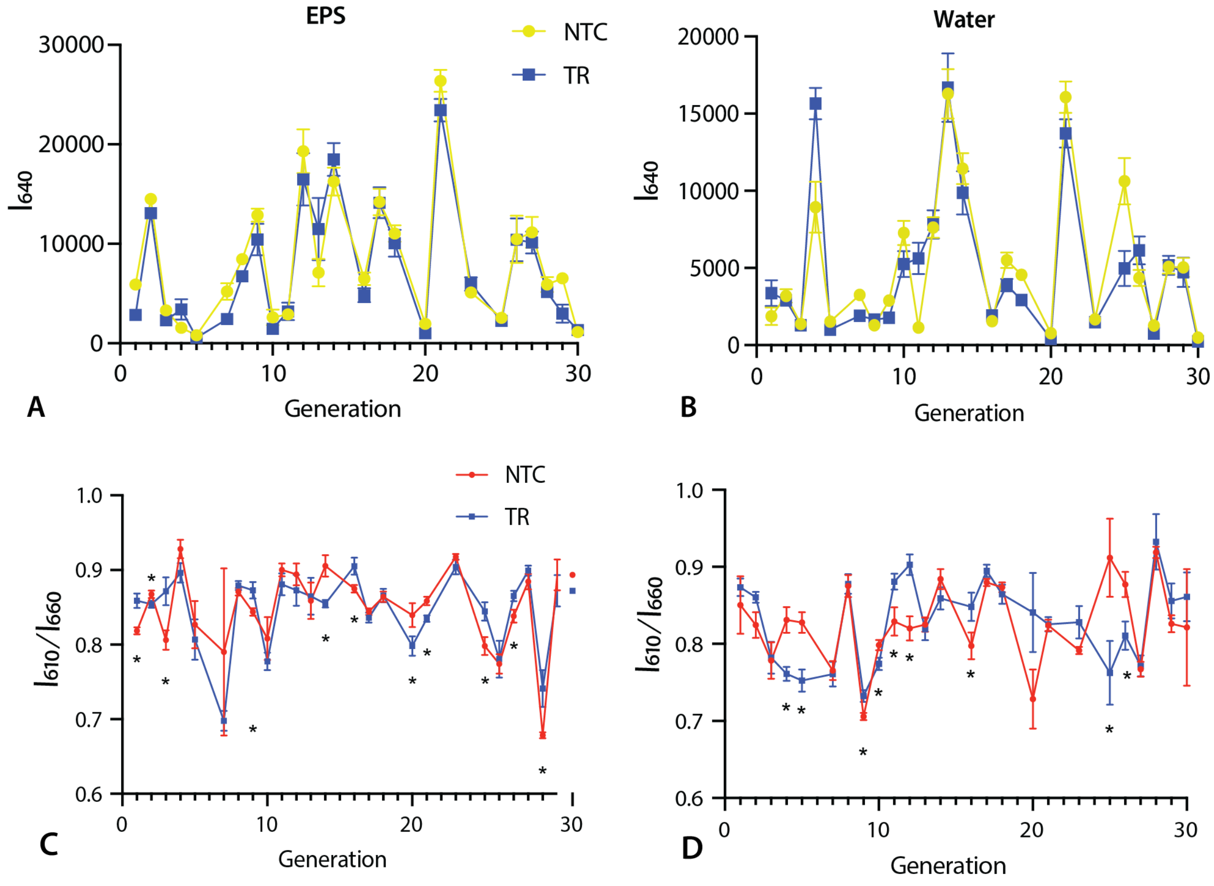

Figure S3. Raw NR fluorescence intensity plots for NTCs and TRs of EPS and water samples with Triton. **A** – I_{640} for EPS samples over 30 generations; **B** – water samples; **C** – I_{610}/I_{660} for EPS; **D** – I_{610}/I_{660} for water. Error bars are standard error (12 replicates).

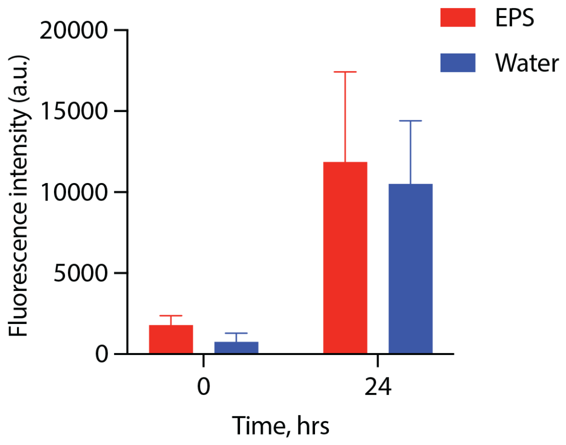

Figure S4. Comparison between Nile Red fluorescence emission spectra with 480 nm excitation and 640 nm emission wavelengths for 20 mM 1:1 DA:DN with 0.05% Triton samples incubated in water or EPS for 0 and 24 hours. Error bars are standard error (16 replicates).

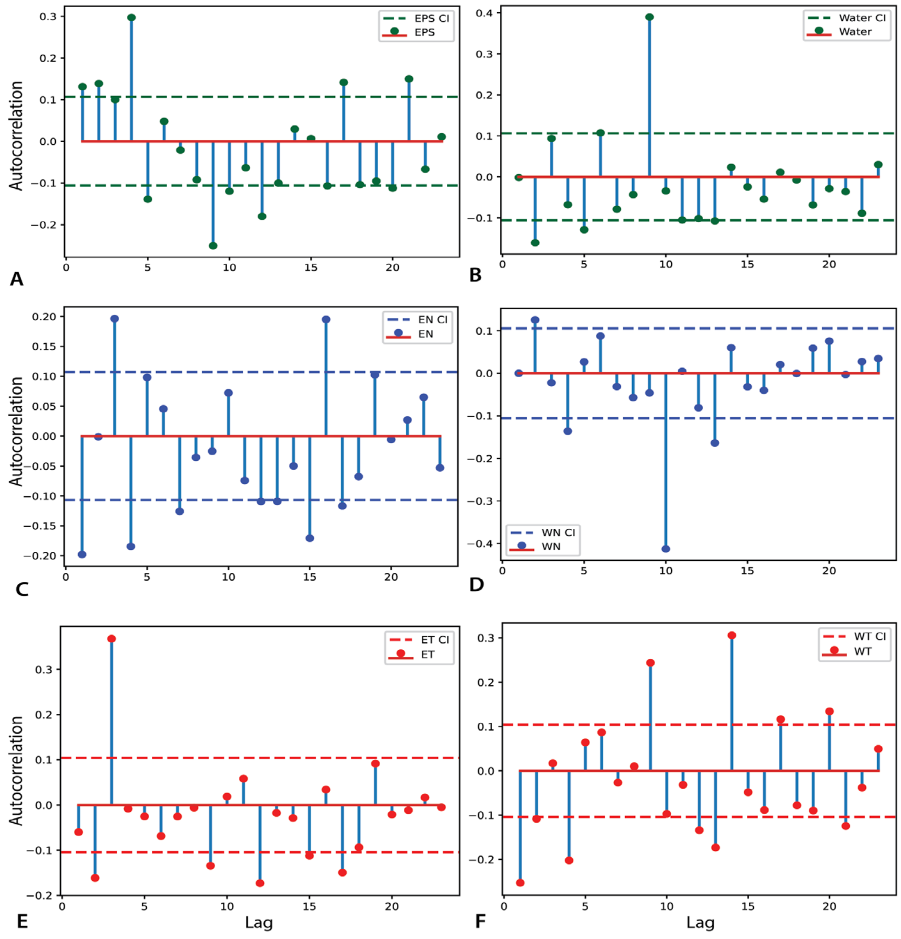

Figure S5. Autocorrelation function (ACF) values for the 12-replicate Triton samples over 25 generations with 95% confidence intervals. **A–B**: ACF plots for the NR fluorescence TR/NTC ratios of EPS (**A**) and water (**B**) samples; **C–F**: ACF plots for the raw NR Fluorescence of EPS NTCs

(**C**), Water NTCs (**E**), EPS TRs (**E**), and Water TRs (**F**). Python code for this analysis is attached separately.

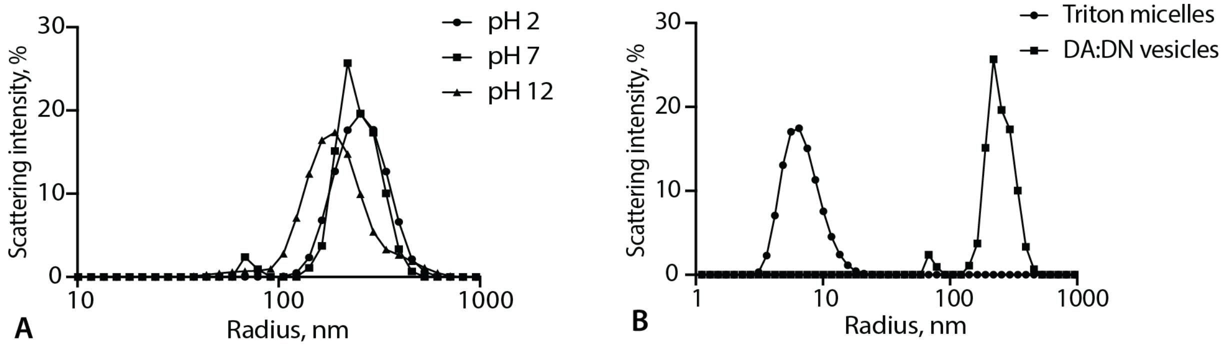

Figure S6. A – size distributions of 20 mM 1:1 DA:DN vesicles at pH 2, 7 and 12 that demonstrates lack of micelles or large particles in acidic and basic samples (1 replicate). **B** – comparison between the sizes of pure 0.05% Triton X-100 micelles and DA:DN vesicles (1 replicate).

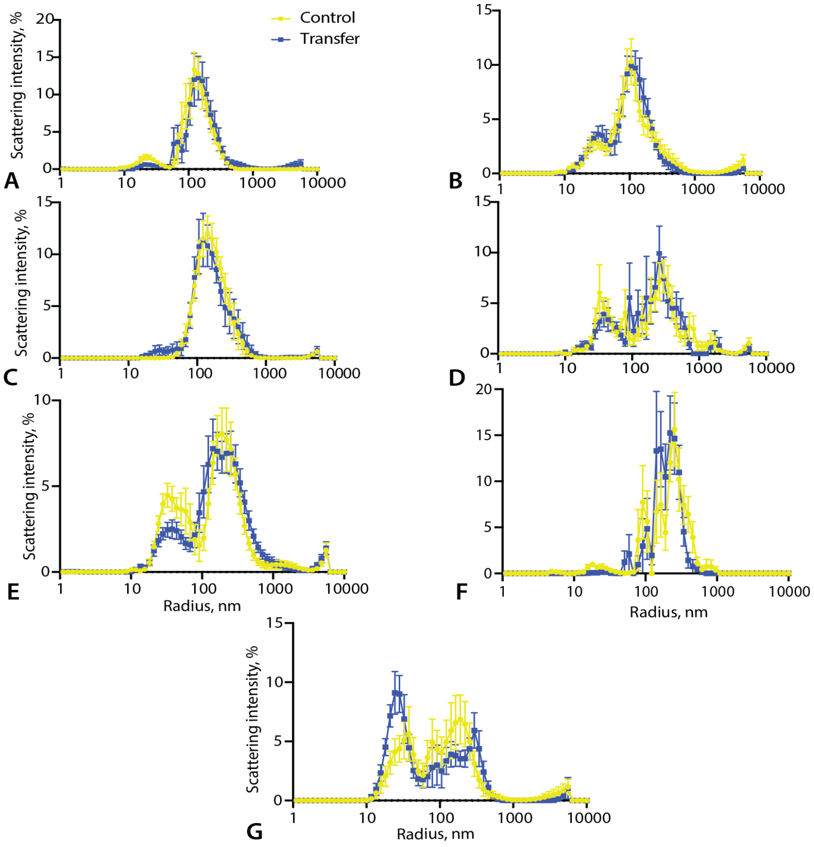

Figure S7. Size distributions of samples containing Triton X-100: **A** – generation 5, EPS; **B** – generation 5, water; **C** – generation 10, EPS; **D** – generation 10, water; **E** – generation 20, water; **F** – generation 25, EPS; **G** – generation 25, water. Error bars are standard error (10 replicates).

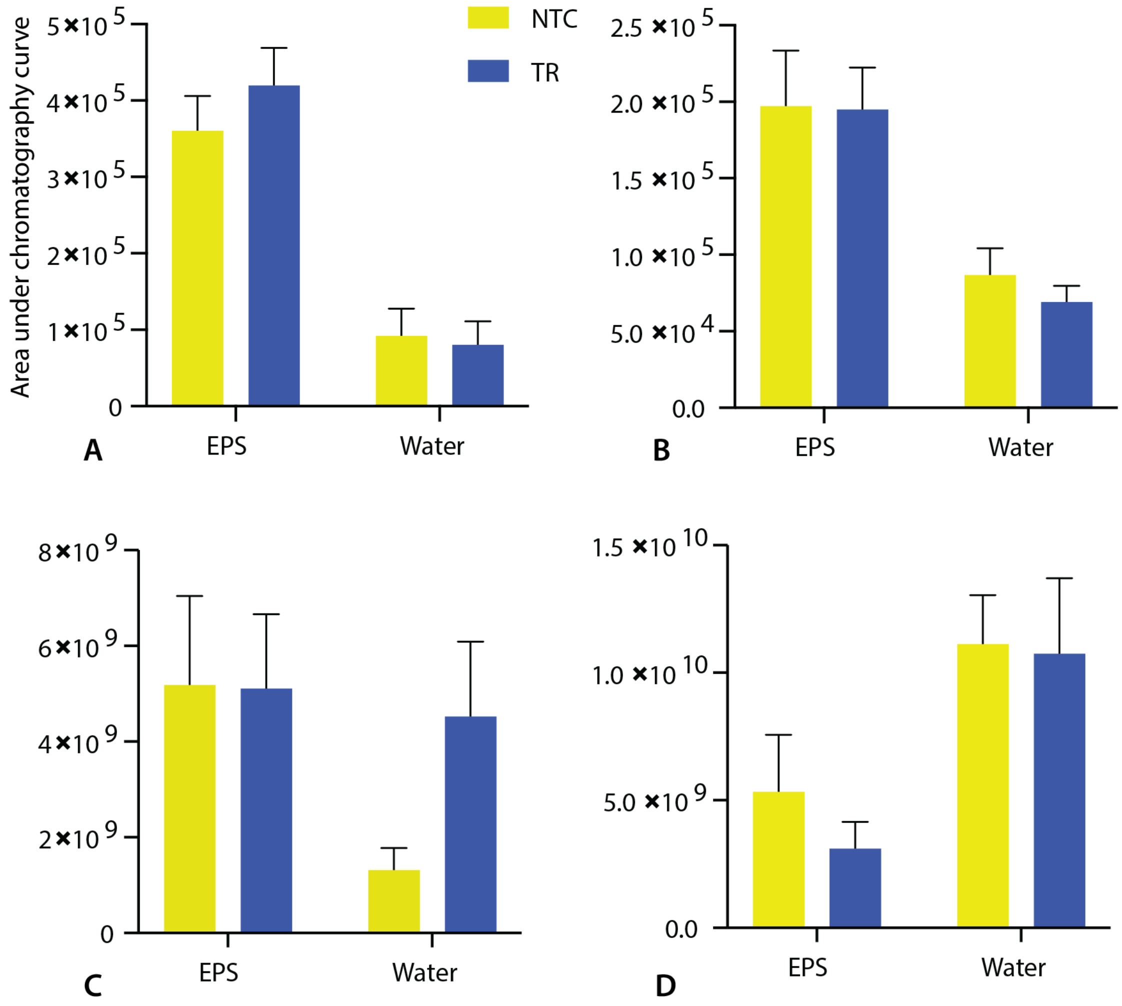

Figure S8. Relative abundance of amphiphiles as measure by LC peak area under the curve for samples without Triton X-100. **A** – DA, generation 25; **B** – DA, generation 30; **C** – DN, generation 25; **D** – DN, generation 30. No significant differences between TRs and NTCs are observed. Error bars are standard error (12 replicates).
